# Supplementary material for: Cellular proliferation biases clonal lineage tracing and trajectory inference
Source: Bioinformatics. 2024 Aug 5;40(8):btae483. doi: 10.1093/bioinformatics/btae483 (PMC11316616; doi:10.1093/bioinformatics/btae483)
Supplement: btae483_Supplementary_Data [file btae483_supplementary_data.pdf]

A. Appendix

A.1. Derivation of biased proportions in MT at the later timepoint t_2

We include below the derivation of probabilities of subpopulation proportions at t_2, which is analogous to the derivation included in the paper for t_1 (Equations 1 and 2). Derivations for time points beyond t_1 and t_2 would follow similar arguments.

As we derive below, the cell type proportions in MT (as defined in Section 2.1) at t_2 are biased by the relative cell growth between t_0 and t_1. Let $m(c)$ denote the number of cells in clone c at t_1, or equivalently, the net growth rate between t_0 and t_1 of the cell at t_0 which was barcoded as clone c. Then we have:

$$\begin{aligned}\mathbb{P}(y \in MT \cap \mathcal{M}(t_2)) &= \mathbb{P}(y \in \mathcal{B}(t_2))\mathbb{P}(\text{at least one cell in } \mathcal{C}(y) \text{ is in } \mathcal{B}(t_1)) \\ &= \mathbb{P}(y \in \mathcal{B}(t_2))(1 - \mathbb{P}(\text{no cells in } \mathcal{C}(y) \text{ are in } \mathcal{B}(t_1))) \\ &= r_{t_2} b \left(1 - \prod_{i=1}^{m(C(y))} (1 - r_{t_1} b)\right) \\ &= r_{t_2} b \left(1 - (1 - r_{t_1} b)^{m(C(y))}\right) \end{aligned} \tag{A.1}$$

and, if we simplify the expression by assuming that all clones of type l have the same value of $m(\cdot)$, which we denote $m(l)$ as in the derivation of Equation 2, then we have

$$\begin{aligned}\mathbb{P}(y \in \mathcal{M}(t_2) \cap MT \text{ and is type } l) &= \mathbb{P}(y \in \mathcal{M}(t_2) \cap MT \mid y \in \mathcal{M}(t_2) \text{ is type } l)\mathbb{P}(y \in \mathcal{M}(t_2) \text{ is type } l) \\ &= r_{t_2} b \left(1 - (1 - r_{t_1} b)^{m(l)}\right) \frac{N_{t_2}(l)}{N_{t_2}},\end{aligned}$$

and therefore we obtain,

$$\begin{aligned}\mathbb{P}(y \in \mathcal{M}(t_2) \text{ is type } l \mid y \in \mathcal{M}(t_2) \cap MT) &= \frac{\mathbb{P}(y \in \mathcal{M}(t_2) \cap MT \text{ and is type } l)}{\mathbb{P}(y \in \mathcal{M}(t_2) \cap MT)} \\ &= \frac{r_{t_2} b \left(1 - (1 - r_{t_1} b)^{m(l)}\right) \frac{N_{t_2}(l)}{N_{t_2}}}{\sum_l r_{t_2} b \left(1 - (1 - r_{t_1} b)^{m(l)}\right) \frac{N_{t_2}(l)}{N_{t_2}}}.\end{aligned} \tag{A.2}$$

These equations are very similar to those for t_1 (Equations 1 and 2) except here the probabilities depend on growth rates between t_0 and t_1 rather than between t_0 and t_2. Comparing Equation A.2 to Equation 2, if the true cell type proportions at t_1 and t_2 are the equal then the bias in the proportions may be lesser or greater at t_1 than at t_2 (depending if $g(l) > 1.0$ or < 1.0 respectively) due to the exponent being $m(l)g(l)$ rather than $m(l)$.

A.2. Derivation of biased proportions in MT for an arbitrary number of time points

We include below the derivation of the probabilities of subpopulation proportions at any sample time $t_k \in \{t_1, t_2, \ldots, t_T\}$ in a time course with a finite but arbitrary number of time points $T \geq 2$. This derivation follows a similar argument to that for t_1 and t_2 for $T = 2$ in Section 2.1 and Appendix A.1 respectively.

Let x denote a cell alive at time t_k and let r_t denote the sampling rate at time $t \in \{t_1, t_2, \ldots, t_T\}$. Then, by the law of total probability and De Morgan's law, we have:

$$\begin{aligned}\mathbb{P}(x \in MT \cap \mathcal{M}(t_k)) &= \mathbb{P}(x \in \mathcal{B}(t_k))\mathbb{P}(\cup_{j=1, j\neq k}^{T}\{\text{at least one cell in } \mathcal{C}(x) \text{ is in } \mathcal{B}(t_j)\}) \\ &= \mathbb{P}(x \in \mathcal{B}(t_k)) \left(1 - \mathbb{P}(\cap_{j=1, j\neq k}^{T}\{\text{no cells in } \mathcal{C}(x) \text{ are in } \mathcal{B}(t_j)\})\right).\end{aligned}$$

Now let $\tilde{g}(C(x), t)$ denote the sum of the growth rates of all cells alive in clone $C(x)$ at time t. Then, by the same independence assumption made in Section 2.1, the probability of the intersection can be broken down as the following product,

$$\begin{aligned}\mathbb{P}(x \in MT \cap \mathcal{M}(t_k)) &= \mathbb{P}(x \in \mathcal{B}(t_k)) \left(1 - \prod_{j=1, j\neq k}^{T} \mathbb{P}(\text{no cells in } \mathcal{C}(x) \text{ are in } \mathcal{B}(t_j))\right) \\ &= r_{t_k} b \left(1 - \prod_{j=1, j\neq k}^{T} \prod_{i=1}^{\tilde{g}(C(x), t_{j-1})} (1 - r_{t_j} b)\right).\end{aligned} \tag{A.3}$$

By Equation A.3, since each factor in bounded in $[0, 1]$ (as they represent probabilities), as T increases the product over the time points will be non-increasing, and hence the size of $\mathbb{P}(x \in MT \cap \mathcal{M}(t_k))$ will be non-decreasing. To reason how this effects the biased proportions of subpopulations we consider the following probability for some cell type (or any subpopulation correlated with growth) l:

$$\mathbb{P}(x \in \mathcal{M}(t_k) \text{ is type } l \mid x \in \mathcal{M}(t_k) \cap MT) = \frac{\mathbb{P}(x \in \mathcal{M}(t_k) \cap MT \;\mid\; x \text{ is type } l)\frac{N_{t_k}(l)}{N_{t_k}}}{\sum_l \mathbb{P}(y \in \mathcal{M}(t_k) \cap MT \;\mid\; x \text{ is type } l)\frac{N_{t_k}(l)}{N_{t_k}}}, \tag{A.4}$$

where $\mathbb{P}(y \in \mathcal{M}(t_k) \cap MT \;\mid\; x \text{ is type } l)$ takes an analogous form to in Section 2.1 based off of Equation A.3. From Equation A.4 and the observation that $\mathbb{P}(x \in MT \cap \mathcal{M}(t_k))$ will be non-decreasing as T increases, the behaviour of the probability representing the biased proportion $\mathbb{P}(x \in \mathcal{M}(t_k) \text{ is type } l \mid x \in \mathcal{M}(t_k) \cap MT)$ as T increases will depend on how the denominator changes. More specifically, since $\frac{N_{t_k}(l)}{N_{t_k}}$ is constant for all l as T increases, it will depend on how the values of $\mathbb{P}(x \in \mathcal{M}(t_k) \cap MT \;\mid\; x \text{ is type } l)$ behave for all types l.

Consider the case in which $\mathbb{P}(x \in \mathcal{M}(t_k) \cap MT \;\mid\; x \text{ is type } l)$ only increases for type l (for all other types the probability stays constant as T increases). Then it is easy to prove with simple algebraic manipulation that the new expression (after the increase in T) on the right-hand side of Equation A.4 is greater than the previous expression. This case demonstrates that the size of the bias effect may increase as T increases beyond $T = 2$. In general, whether the bias effect will increase depends on complex interactions between the growth rates in the new periods of time in the time course, as summarized with Equation A.4. This equation may be used to estimate the size of the bias effect as demonstrated using Equation 2 in Section 2.1.

B. Appendix

B.1. *CoSpar-MT* and *LineageOT-MT* Results Across Simulated Datasets

This section contains the fate probability estimation results from *CoSpar-MT* and *LineageOT-MT* on the two simulation scenarios discussed in the manuscript (see Sections 2.2 and 2.3). The results are shown in Figure 1.

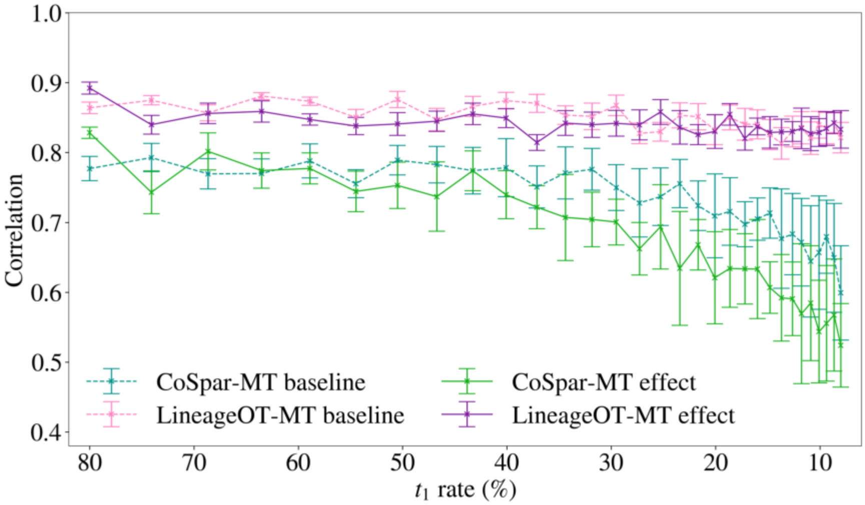

(a) Simulation 1 - from Section 2.2

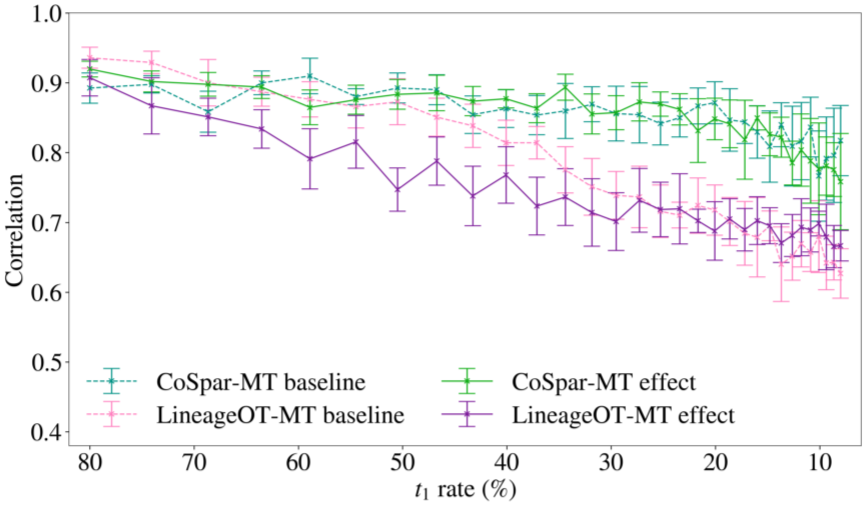

(b) Simulation 2 - from Section 2.3

Fig. 1: **Performance of *CoSpar-MT* and *LineageOT-MT* for both simulation scenarios:** Pearson correlation coefficient between the true fate probability and the fate probability estimated from *CoSpar-MT* and *LineageOT-MT* as a function of t_1 rate (combined sampling and barcode rate $r_{t_1}b$) for $b = 0.8$ fixed for the *baseline* and *effect* cases. Values are summarized as a mean across ten replicates with error bars given by the standard deviation. The plot shows that *CoSpar-MT* has a larger difference in performance between *baseline* and *effect* for Simulation 1, while *LineageOT-MT* shows a more significant performance decrease for Simulation 2. Results are identical for fate B and hence they have been omitted.

B.2. *CoSpar-ST* and *LineageOT* Results Across Simulated Datasets

This section contains the fate probability estimation results from *CoSpar-ST* and *LineageOT* on the two simulation scenarios discussed in the manuscript (see Sections 2.2 and 2.3). The results are shown in Figure 2. For both methods and simulation scenarios the correlation in the *effect* case is higher or equal to the *baseline*, demonstrating no significant negative effect in the presence of the bias. Recall that this is the expected outcome since single-time information is not biased.

Figure 2 shows significantly higher performance in the *effect* case for some sampling rates and for *CoSpar-ST* in particular. A slight increase in performance may be explained by the slightly higher (0.08-1.5%) t_2 sampling rate in the *effect* case (caused by the increased growth and sampling a fixed number of cells). The significantly higher correlation for *CoSpar-ST* in the *effect* cases may indicate that the method does more poorly in scenarios with equal growth rates, perhaps as cells cannot be differentiated by their growth rates.

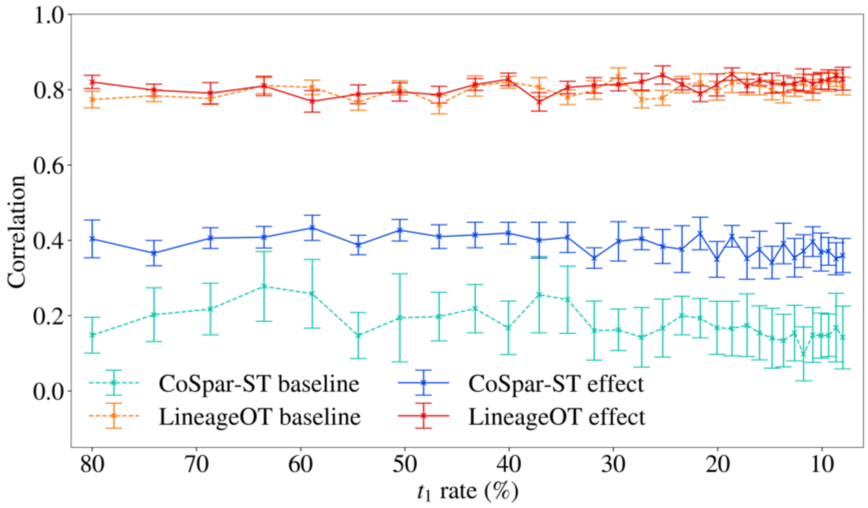

(a) Simulation 1 - from Section 2.2

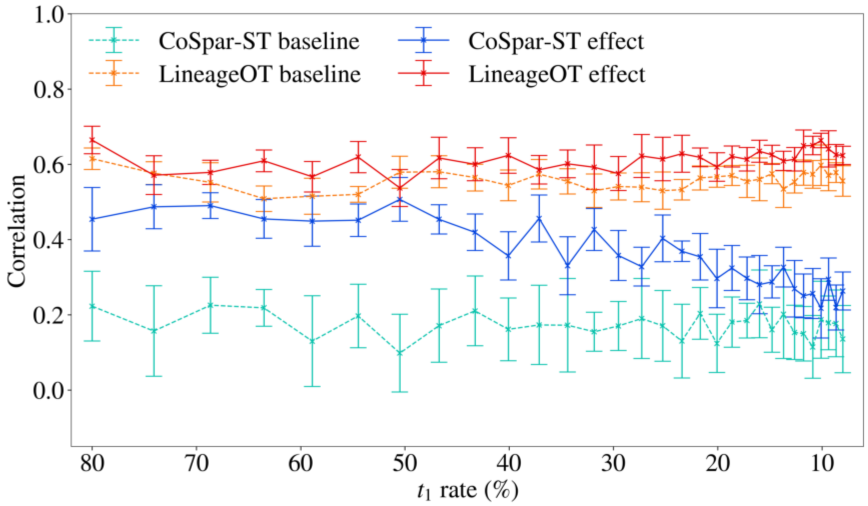

(b) Simulation 2 - from Section 2.3

Fig. 2: **Single-time methods are not negatively impacted by the bias:** Pearson correlation coefficient between the true fate probability and the fate probability estimated from $CoSpar\text{-}ST$ and $LineageOT$ as a function of t_1 rate (combined sampling and barcode rate $r_{t_1}b$) for $b = 0.8$ fixed for the *baseline* and *effect* cases. Values are summarized as a mean across ten replicates with error bars given by the standard deviation. These two methods, that incorporate only single-time clonal information, exhibit equal or better fate probability estimation performance in the *effect* case (in the presence of the bias) over the *baseline* case. The significantly higher correlation for $CoSpar\text{-}ST$ in the *effect* cases may indicate that the method does more poorly in scenarios with equal growth rates, perhaps as cells cannot be differentiated by their growth rates. Results are identical for fate B and hence they have been omitted.
